# Sprawl: The New Manifest Destiny?

**DOI:** 10.1289/ehp.112-a620

**Published:** 2004-08

**Authors:** Charles W. Schmidt

Seen from 400 miles above the Earth, the greater Washington, D.C.– Baltimore area is an aggressive consumer of farmland and open spaces. Computer-enhanced satellite images of the area show paved surfaces as crimson tentacles, pushing steadily out from the urban core. Recent studies by the National Aeronautics and Space Administration now suggest the land area occupied by Washington, D.C., and surrounding communities will expand 80% over its current size by 2030.

Urban sprawl so extensive that you can watch it from space is hardly limited to the nation’s capital. Indeed, sprawl—defined as low-density development that outpaces population growth—is endemic throughout much of the United States. Donald Chen, executive director of Smart Growth America, a nonprofit research coalition in Washington, D.C., says that the overall declines in urban density, loss of open spaces, and increased auto use that accompany sprawl are continuing “virtually unabated.” Those who leave cities for the suburbs may expect a healthier, cleaner environment, but sprawl developments actually present a range of health risks including poor air quality from rising vehicle use, watershed pollution, and a built environment that limits opportunities to walk from homes to businesses and schools, thereby exacerbating obesity and related medical problems, such as heart disease.

Sprawl first surfaced as a federal policy issue in the late 1990s, driven mainly by then–vice president Al Gore, who made it a centerpiece of his environmental platform. Researchers were increasingly aware that sprawl was a growing problem fraught with economic, ecologic, and, possibly, health consequences. However, these consequences were not well understood, says Reid Ewing, an associate and research professor at the University of Maryland National Center for Smart Growth Research and Education. “Sprawl was mainly a political issue back then,” he recalls. “There were various hypotheses about the magnitude of sprawl and its impacts, but sprawl had been neither measured in a sophisticated way nor related objectively to a range of outcomes such as loss of farmland and increased air pollution.”

Since the turn of the millennium, Ewing says, numerous studies have sought to quantify sprawl, define its causes, and investigate its health and environmental concerns. At the same time, alternatives to sprawl have been studied and applied in many areas, with varying levels of success.

## Defining Sprawl and Its Effects

During the 1990s, there was no consistent definition for sprawl. Experts compared it to obscenity: hard to define, but obvious when you see it. But several years of focused study have since cleared up confusion over what sprawl actually is. In its groundbreaking 2002 report titled *Measuring Sprawl and Its Impact*, Smart Growth America defined sprawl as the outcome of four related factors: low residential density; a poor mix of homes, jobs, and services; limited activity centers and downtown areas; and limited options for walking or biking. This report—the first to create a multidimensional picture of sprawl and its effects—ranked 83 metropolitan areas according to a “sprawl index” derived from 22 separate measures based on the four factors described above. According to this ranking, Riverside–San Bernardino, California, about 60 miles east of Los Angeles, is the most sprawling metropolitan area in the country, while New York City is the least.

Along with a greater understanding of sprawl’s defining features has come improved knowledge of its related health hazards. For instance, the Smart Growth America report showed that sprawl correlated directly with rising vehicle use. The finding was based on a comparison of each city’s overall sprawl index and a parameter known as vehicle-miles traveled (VMT) per person (which Ewing has found is also a risk factor for crashes and traffic fatalities; for more on the growing problem of traffic crashes, see “Vehicular Manslaughter: The Global Epidemic of Traffic Deaths, p. A628 this issue). VMT can be derived from data gathered by the U.S. Department of Transportation. The correlation between sprawl and VMT is small, the report states, but sufficient to produce significant increases in vehicle emissions across metropolitan regions.

Among the most problematic vehicle emissions are nitrogen oxides (NO_x_), a group of highly reactive combustion gases. Automotive controls have lessened emissions of other pollutants, but NO_x_—because of its chemical properties—is still emitted at high levels. This is unfortunate because NO_x_ combines with airborne particles and sunlight to form ground-level ozone, a toxic chemical with dangerous respiratory effects, especially among children, those with asthma, and the elderly.

“There doesn’t seem to be any doubt that sprawling metro areas have worse ozone pollution than more compact areas,” says Ewing. Data gathered by Smart Growth America show that high ozone levels are tightly linked to sprawl development. In fact, high-density areas were found to have ozone levels that averaged 51 parts per billion less than low-density areas; the U.S. Environmental Protection Agency (EPA) standard for ambient ozone is 80 parts per billion, averaged over an eight-hour period.

These results may appear at odds with common sense; after all, shouldn’t automotive pollution be worse in urban areas than in outlying communities? “You would think you’d have less congestion and cleaner air in the suburbs,” Ewing concedes. “But people drive so much more in sprawling areas that they offset the benefits of dispersal. We found ozone levels were higher and congestion was about the same, largely due to these offsetting effects.”

With its focus limited to ozone, *Measuring Sprawl and Its Impact* is silent on other automotive pollutants that may also elevate health risks. However, the Sierra Club recently conducted a broad investigation of highway health risks from polluted air, emphasizing in particular the role of carcinogenic hydrocarbon emissions from cars and trucks. The organization’s 2004 report, titled *Highway Health Hazards*, compiled the results of 24 academic studies published in peer-reviewed journals such as *JAMA*, *The Lancet*, and *EHP*, among others. These studies linked traffic-related air pollution to health problems such as asthma, cancer, premature birth, low birth weight, and a generally higher risk of death among residents who lived near busy roadways, particularly those roads carrying more than 150,000 vehicles per day.

Brett Hulsey, a transportation expert at the Sierra Club, says the findings reinforce the view that vehicle emissions and health effects are related. “Some of the worst air pollution is in the car itself,” Hulsey explains. “People who drive for hours every day are stuck in a plume of cancer-causing chemicals [spewing from the cars around them]. So, what we’re saying is that more sprawl equals more driving, and that more driving equals greater health risk. Therefore, sprawl and health risks are related.”

## A Focus on Obesity

In a recent development, sprawl researchers have also begun to address the built environment’s influence on physical activity and obesity. The obesity epidemic in the United States and other countries throughout the world is now viewed as a growing public health crisis. Both child and adult obesity rates in the United States have doubled since 1980, according to the Centers for Disease Control and Prevention. The expanding waistline is a major factor in the rise of type 2 diabetes mellitus, which also has achieved epidemic proportions, affecting some 17 million Americans, according to the National Institute of Diabetes and Digestive and Kidney Diseases. Add cardiovascular disease, low self-esteem, and depression to the list of related health problems, and obesity will soon surpass smoking as the nation’s leading health threat, experts say.

Hypothesized links between the built environment and obesity are now being explored jointly by experts in planning, nutrition, and public health [see “Fighting Obesity Through the Built Environment,” p. A616 this issue]. This multidisciplinary union has produced important new evidence suggesting that sprawl and obesity are likely related. A study published in the August 2004 issue of the *American Journal of Preventive Medicine* related body mass to measures of sprawl within a one-kilometer distance of each participant’s residence. The study, led by Lawrence Frank, an associate professor of community and regional planning at the University of British Columbia, focused on 10,898 residents of Atlanta, Georgia, a city that ranks fourth on Smart Growth America’s top-10 list of the most sprawling U.S. metropolitan areas.

Frank’s results showed that sprawl development was associated with both increased time spent in cars and increases in body weight. Specifically, for every extra 30 minutes of commuting time per day, participants had a 3% greater likelihood of obesity than peers who drove less. The study also found that people who lived within walking distance (defined as a half-mile) of shops were 7% less likely to be obese than counterparts who lived farther away. “These findings are intuitively obvious,” Frank says. “But now we actually have the data to back them up.”

Frank is currently doing another study in which subjects wear accelerometers, which measure motion. This yields data on activity patterns, which he and colleagues will correlate with obesity and residential land use features. Data analysis is preliminary, but correlations between activity and residential land use features observed thus far are “very strong,” he says.

Frank’s research, with its kilometer-scale resolution, builds on an earlier study by Ewing and colleagues, published in the September/October 2003 issue of the *American Journal of Health Promotion*. This study showed that urban design at the county level in Atlanta also correlated with physical activity and obesity. When it was released, the study triggered widespread media coverage; it provided the most compelling evidence to date that sprawl promotes obesity by fostering a sedentary lifestyle. Specifically, the study showed that those who lived in sprawling counties were likely to walk less, weigh more, and have greater prevalence of hypertension than those living in more compact counties.

Ewing and Frank caution that the current evidence doesn’t conclusively establish a cause–effect relationship between sprawl and obesity. Other variables are also at play, chief among them the types of food available locally and the calories consumed compared to those expended. Furthermore, current evidence derives from cross-sectional studies that merely provide snapshots of weight and behavior at single time points. Longitudinal studies that track participants as they move in and out of sprawling areas are needed to bolster cause–effect hypotheses, Ewing says. “Right now, it’s not clear that sprawl makes people less active,” he explains. “It may be that people who are already less active choose sprawl development as a place to live.”

In choosing low-density development, sprawl inhabitants may also seek a greater connection with nature. But sprawl tends to highly disturb the natural environment. Michael Klemens, a senior conservationist with the Bronx Zoo–based Wildlife Conservation Society and coauthor of the book *Nature in Fragments: The Legacy of Urban Sprawl* (in press), has studied sprawl’s effects on biodiversity in the New York City metropolitan area for more then 25 years. His research, based on field observations and more than 100 years of existing baseline data, shows that 75% of plant and animal species impacted by sprawl in New York are in decline. A residual 25% of species experience population increases, he says, but these tend to be so-called weed species that are able to thrive in fragmented habitats.

Declines in biodiversity have far-reaching ecological impacts. “The gene pool is much smaller, so the system itself is at greater risk,” Klemens explains. “An ecosystem that contains just twenty-five percent of the original flora and fauna is less resilient to change.” Furthermore, he adds, some weed species are competent vectors for disease transmission. White-footed mice, for instance, which thrive in sprawl developments, carry Lyme disease and West Nile virus. Thus, sprawl also contributes to the spread of infectious illnesses, with serious public health effects.

## Real-World Solutions

The chief development alternative to emerge in response to sprawl is “smart growth.” With its focus on urban revitalization and expanded transit options, smart growth seeks to make existing communities places that people want to live. The term was popularized by Parris N. Glendening, governor of Maryland from 1994 to 2002, who in 1997 launched the Smart Growth and Neighborhood Conservation Program to limit sprawl in his state. Today, dozens of environmental groups, civic organizations, and government agencies promote smart growth principles as part of their sprawl reduction programs. These principles include, among other concepts, the promotion of mixed land uses and the creation of attractive neighborhoods with a strong sense of “place,” or local identity and character, where residents can walk freely to the places they need to go.

The Smart Growth Network is a partnership between the EPA and a number of nonprofit, public, and governmental organizations working together to raise public awareness and promote smart growth principles. In its popular first volume of the manual *Getting to Smart Growth*, released in 2001 (a second volume was released in 2003), the Smart Growth Network suggested that towns should return to the designs of the early twentieth century. In those earlier times, land uses were more integrated, enabling people to walk to the corner store, to work, or to school. Today, such uses are more often placed so far apart they can only be reached by car. Numerous communities have sought to reverse this trend.

Portland, Oregon, is an oft-touted model of sprawl containment. The city established an “urban growth boundary” in 1980 that protects nearby farmland surrounding the city and tightly limits development in outlying areas. Portland’s approach has not been without controversy. For several years, the urban growth boundary was accompanied by skyrocketing housing costs and discontent among those who resented restrictions on development. But the high costs of housing—which are in fact attributable to a host of factors, including a high rate of migration to Portland from other states, particularly California—have since declined to the point that they are roughly equivalent to those of other West Coast cities, says Mary Volm, spokesperson for the City of Portland Office of Transportation.

Because of the urban growth boundary, Volm says, Portland has successfully assimilated a sharply rising population without encroaching on its valuable land resources. “We make solid investments to create lively districts and neighborhoods that people are attracted to,” she explains. Portland’s urban designs provide affordable and accessible public transit located close to schools, businesses, and residential communities. In addition, walking and bike paths connect the entire community, which is infused with a multitude of parks and green spaces.

Urban growth boundaries are but one tool among many to limit sprawl. Others include establishing more mixed-use areas (so residents can shorten or eliminate some trips) and creating more density in places that already have or could have transit services. Atlanta began its Livable Centers Initiative (LCI) in 1999 after a 13-county region surrounding the city fell out of compliance with the Clean Air Act. This major program committed $350 million toward alternative transportation projects in surrounding communities that plan for mixed land uses, affordable housing, and increased transportation efficiency. A total of 51 communities have been funded for planning under the program thus far.

Past experience in Atlanta permits an optimistic outlook. In preparation for the 1996 Summer Olympics, the city bolstered public transportation and other traffic control measures in part by substantially increasing service on the rail transit system and making major areas off-limits to vehicular traffic. Once these changes were in place, acute childhood asthma attacks fell by 44%, ozone concentrations fell by 28%, and morning peak traffic fell by 22.5%. These results are described in the 21 February 2001 issue of *JAMA* in a study by Michael Friedman, an epidemiologist at the Centers for Disease Control and Prevention, and colleagues. Thomas Weyandt, director of comprehensive planning with the Atlanta Regional Commission, says the experience also showed that “if you provide more transit, people will use it.”

## And Still There Is Sprawl

Despite growing knowledge of its impacts and an array of development alternatives, sprawl continues to spread, leaving polluted resources and more sedentary populations in its wake. Why? Numerous factors drive the trend. First are the government subsidies that pay for sprawl. Rural roads are built and maintained with twice the federal funding that is devoted to urban road maintenance, according to the Surface Transportation Policy Project, a Washington, D.C.–based nationwide coalition that studies transportation issues. Gasoline, too, is heavily subsidized by the federal government—if the costs of air pollution and protection of national petroleum interests were incorporated into fuel pricing, then gas at the pump would be twice as expensive as it is now, according to the Surface Transportation Policy Project.

A sustained surge in the housing market has also played a significant role. Middle- to upper-middle-class citizens continue to flock to the suburbs in search of safe, affordable housing. Moreover, smart growth projects often conflict with local zoning codes that impede urban revitalization. These laws reflect decades-old efforts to segregate housing from industrial polluters that are rarely found in residential areas today, since heavy industry is no longer the primary engine of the economy. Variances for new urban development can take months or years to process; meanwhile, adequate parking, emergency response, and other related development issues required for urban renewal collapse into a morass of red tape. “A lot of developers just don’t want to fight that battle,” says Jessica Cogan Millman, deputy director of the Smart Growth Leadership Institute, a nonprofit project within Smart Growth America.

Perhaps the greatest barrier to smart growth is the diversity and number of stakeholders required to move the process forward, adds Geoffrey Anderson, director of the EPA Development, Community, and Environment Division. “The whole system is burdened with inertia,” he explains. “You have to interest private-sector developers, you need to secure financing, you need the government to issue permits, and you have to convince residents that well-designed density is in their best interests. At a fundamental level, smart growth requires all these stakeholders to work together. But that doesn’t usually happen. Instead, the system puts out the easiest and most familiar product: development that segregates housing and business and invests little into existing communities—in short, development that is land-consumptive and auto-dependent.”

Weyandt agrees that successful coordination under the LCI has depended on the engagement of local leadership and the extent of community involvement. He, too, points to the challenges raised by logistical issues, particularly zoning ordinances that stand in the way of the process. “Zoning has the perverse effect of discouraging what we want most,” he says. “We’ve looked at these ordinances to see how they stack up against smart growth principles, and it’s not a good record. Sometimes these communities have to amend ordinances before they can get funded under the LCI.” Public buy-in on the process can also pose challenges, Weyandt says.

But with a sensitive, well-prepared approach, planners can convince residents that urban revitalization is good for the city and ultimately good for their health. “Once you start talking about housing density at eighty [dwellings] per acre, some people are going to see that in a negative way,” Weyandt concedes. “But if you see a development that’s not only mixed-use and high-density but also pleasant and attractive, then maybe you can imagine yourself living there.”

In fact, Weyandt says, in-town housing is booming in Atlanta. “Our experience shows that the market responds positively to smart growth options,” he says. “We see this as a long-term process. We facilitate decisions at the local level and reward those who do well. And as for those that aren’t interested, perhaps in a few years they’ll change their minds.”

## Figures and Tables

**Figure:**
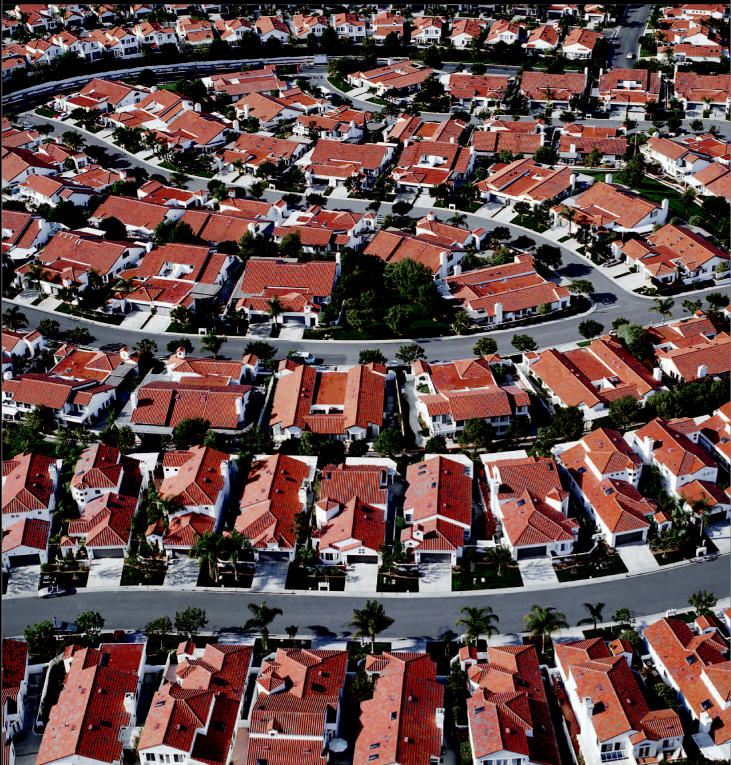


**Figure:**
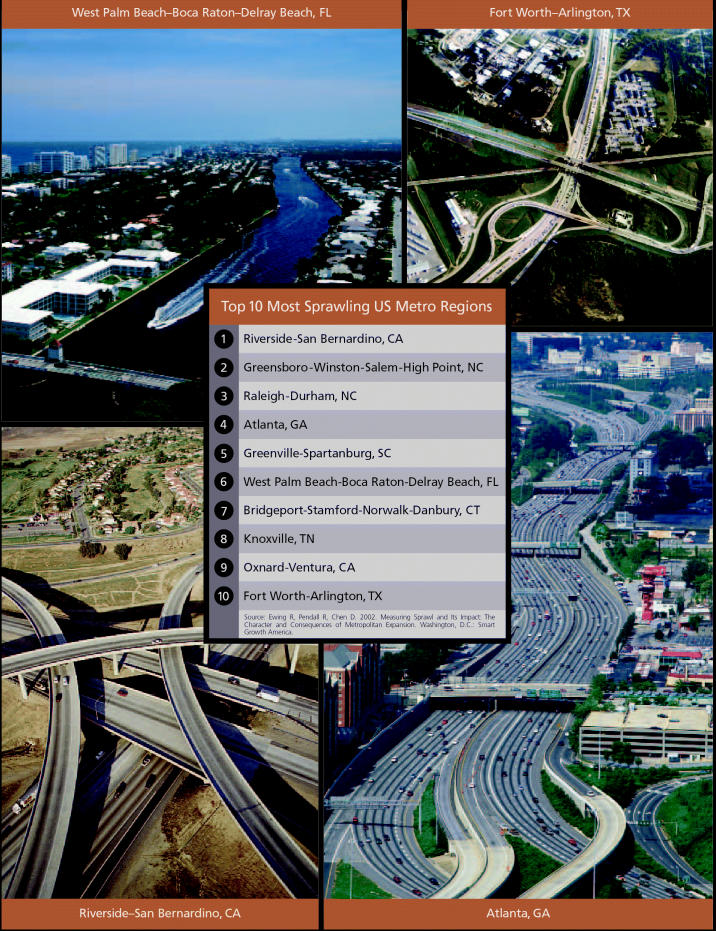


**Figure:**
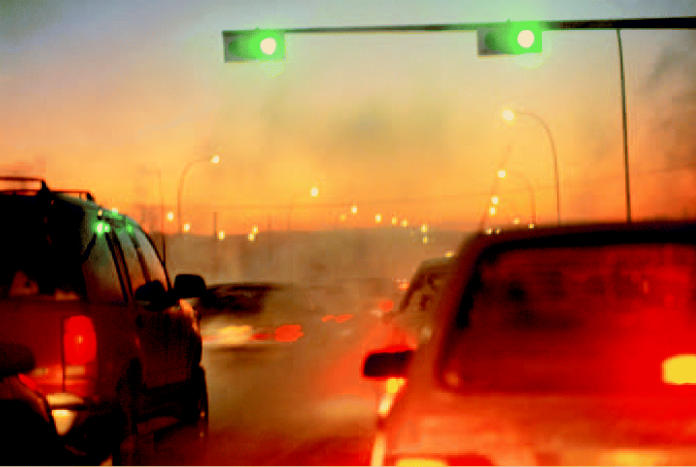
**Mass exodus.** A long line of taillights heading into the dusk as commuters leave the city for the suburbs is an increasingly common sight in metropolitan regions throughout the United States.

**Figure:**
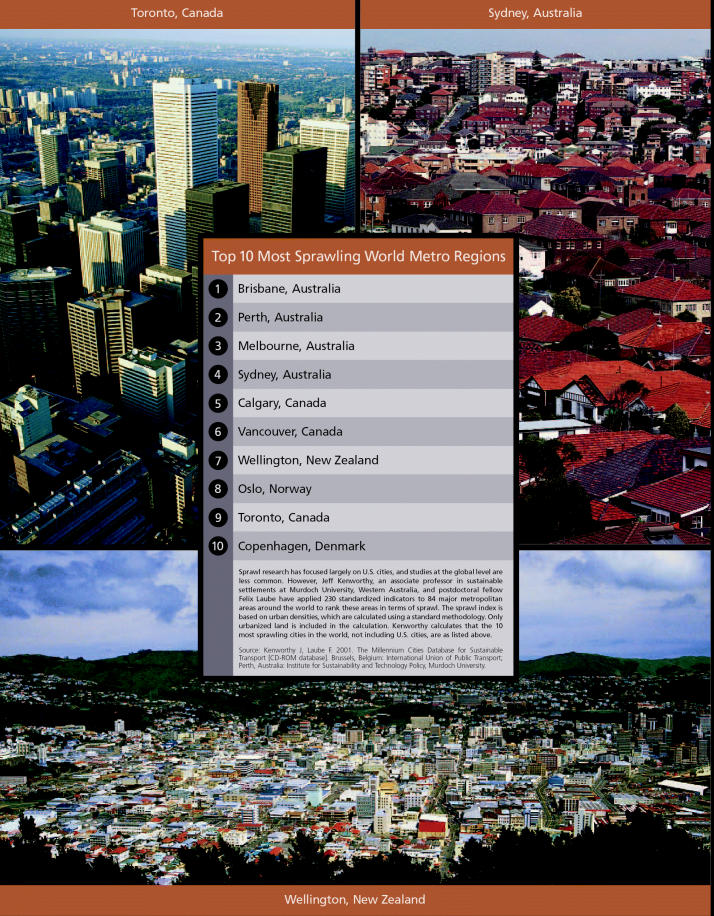


**Figure:**
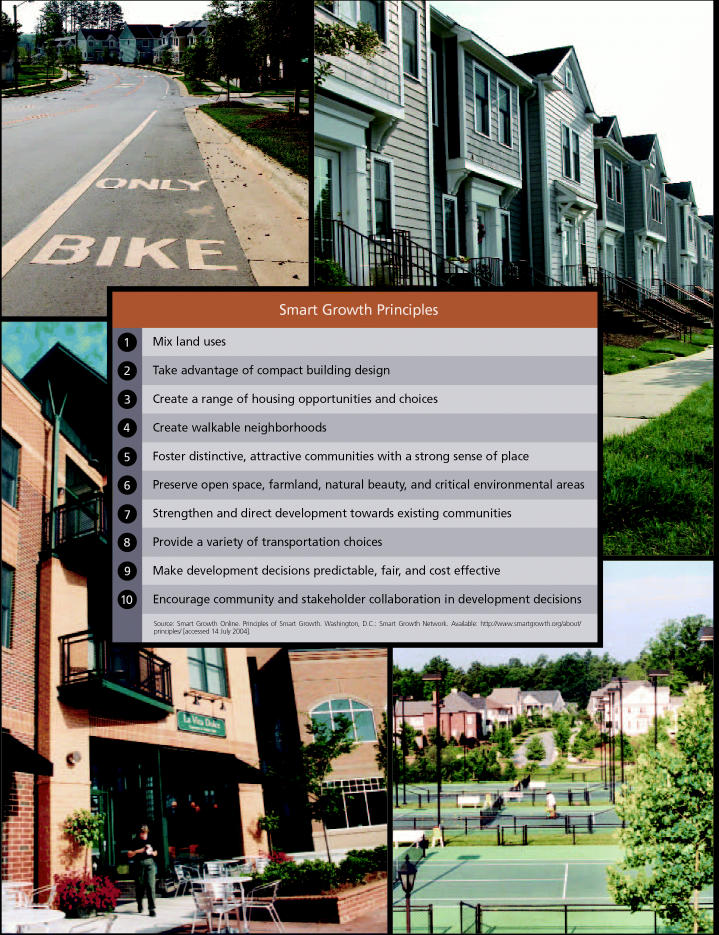


**Figure:**
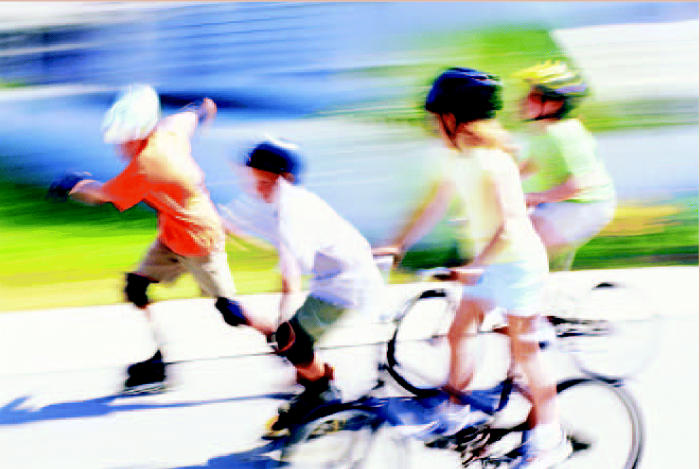
**One sprawl solution.** Suburban communities that encourage and support having an active lifestyle are one part of the answer to the health problems associated with sprawl.

